# Juvenile dermatomyositis associated to lymphoepithelioma, a nasopharyngeal carcinoma

**DOI:** 10.1186/1546-0096-9-S1-P56

**Published:** 2011-09-14

**Authors:** BERG Bica, CT França, AB Vargas

**Affiliations:** 1 Department of Internal Medicine, Hospital Universitário Clementino Fraga Filho, Universidade Federal do Rio de Janeiro, Brazil; 2 Department of Pediatrics, Hospital Municipal da Piedade, Rio de Janeiro, Brazil; 3 Internal Medicine, Universidade Estadual do Rio de Janeiro, Brazil

## Background

Juvenile Dermatomyositis (JDM) is a systemic autoimmune inflammatory muscle disorder and vasculopathy that affects children younger than 16 years old. JDM was not associated with development of malignancies, unlike adult dermatomyositis. Nasopharynx carcinomas (NPC) are rare tumors, corresponding to 2% of tumors of the head and neck and 0,25% of all tumors. They are closely related to the Epstein Barr virus, and also genetic, environmental and viral factors. They occur predominantly among Asians (in certain regions inhabited by Chinese), males and in the range of 40 to 50 years of age. We report a 13-year-old female patient with juvenile DM associated with NPC. The relationship between Epstein-Barr virus infection and both DM and NPC suggests a possible viral cause. Our patient had an undifferentiated carcinoma associated with Epstein-Barr virus.

## Case report

We describe a 13 yo female patient with JDM and lymphoepithelioma of the cavum, a rare nasopharyngeal carcinoma. She presented amyophatic dermatomyositis one year before, with disease well controlled with 10 mg/day oral prednisolone, 12,5 mg weekly oral methotrexate and 400 mg/day hydroxychloroquine. She was affected by 2 months history of disabling pulsing left headache, mainly at noon, with mild relief with analgesics, no irradiations, no unleashing or worsening events. After one week, she presented bloody epistaxis, mainly at morning that ceased spontaneously after 10 days. She had purulent rhinorrhea, left cervical pain associated with a neck mass at the angle of the left jaw and fever, well controlled with antipyretics. She was first treated with antibiotics for 14 days (amoxicillin) for bacterial sinusitis, but only the fever and the purulent aspect of the rhinorrhea improved. The fever and local pain persisted. A CT Scan of the cranial region was performed and another sequence of antibiotics was prescribed (amoxicillin-clavulanate) without improvement. Finally she was biopsied and the diagnosis of lymphoepithelioma. She is being treated with chemotherapy and radiotherapy.

## Conclusion

Although the association of JDM and malignancies is not as common as in adult dermatomyositis, malignancies can occur in patients with JDM. This case alert we that malignancies could be not so uncommon as formerly described, and this association carries a poor prognosis.

